# Introgression of a synthetic sex ratio distortion system from *Anopheles gambiae* into *Anopheles arabiensis*

**DOI:** 10.1038/s41598-019-41646-8

**Published:** 2019-03-26

**Authors:** Federica Bernardini, Antonios Kriezis, Roberto Galizi, Tony Nolan, Andrea Crisanti

**Affiliations:** Department of Life Sciences, Imperial College London, South Kensington Campus, London, SW7 2AZ UK

**Keywords:** Molecular biology, Biotechnology, Molecular engineering

## Abstract

I-PpoI is a homing endonuclease that has a high cleavage activity and specificity for a conserved sequence within the ribosomal rDNA repeats, located in a single cluster on the *Anopheles gambiae* X chromosome. This property has been exploited to develop a synthetic sex ratio distortion system in this mosquito species. When I-PpoI is expressed from a transgene during spermatogenesis in mosquitoes, the paternal X chromosome is shredded and only Y chromosome-bearing sperm are viable, resulting in a male-biased sex ratio of >95% in the progeny. These distorter male mosquitoes can efficiently suppress caged wild-type populations, providing a powerful tool for vector control strategies. Given that malaria mosquito vectors belong to a species complex comprising at least two major vectors, we investigated whether the sex distorter I-PpoI, originally integrated in the *A*. *gambiae* genome, could be transferred via introgression to the sibling vector species *Anopheles arabiensis*. In compliance with Haldane’s rule, F1 hybrid male sterility is known to occur in all intercrosses among members of the *Anopheles gambiae* complex. A scheme based on genetic crosses and transgene selection was used to bypass F1 hybrid male sterility and introgress the sex distorter I-PpoI into the *A*. *arabiensis* genetic background. Our data suggest that this sex distortion technique can be successfully applied to target *A*. *arabiensis* mosquitoes.

## Introduction

Since the beginning of the 21^st^ century, the number of deaths caused by malaria has nearly been halved. Nevertheless, estimates remain dramatically high, reportedly reaching close to half a million in 2017^1^. Insecticide-treated bed nets, indoor residual spraying and artemisinin-based combination drug therapies have played a major role in the reduction of mortality and morbidity due to malaria. However, these traditional methods have failed to achieve a reduction in malaria prevalence since 2016^1^. A successful intervention towards disease eradication requires novel and more efficient technologies that are affordable and sustainable. In this regard, a number of genetic strategies that focus on the control of the mosquito vector have been developed. Among these are sex distortion approaches which rely on the induction of a male-biased sex ratio as a means to suppress or eliminate target populations^2,3^. One such system has been validated in *Anopheles gambiae* where it generated offspring with a >95% sex bias towards males^4^. The technique focuses on the activity of endonucleases such as I-PpoI. This enzyme has high cleavage specificity for a conserved sequence within the ribosomal DNA repeats located in a single cluster on the *A*. *gambiae* X chromosome^5–7^. The expression of the endonuclease during meiosis in transgenic male mosquitoes causes the shredding of the X chromosome leaving only Y chromosome-bearing sperm viable^8^. A similar result is achieved when a CRISPR-based nuclease is used to target the same rDNA sequence^9^. Transgenic males carrying an autosomal insertion of the synthetic sex distorter, if released in sufficient numbers, can effectively suppress caged wild-type populations, thus constituting a promising tool for vector control strategies. Furthermore, mathematical modelling indicates that this approach would be more efficient than other technologies such as Sterile Insect Techniques (SITs)^4^.

Malaria mosquito vectors belong to a complex that includes at least seven sibling species with varying levels of range overlap and reproductive isolation^10^. Due to their wide geographic distribution and marked anthropophily, *A*. *gambiae* and *Anopheles arabiensis* represent the most important vectors of human malaria^11,12^. Genetic flow between these species is limited by both pre- and post-zygotic isolation mechanisms. Cues used for species recognition are still unclear but they might involve differences in the wing beat frequencies, swarming behaviour and odour signals in the two species^13–18^. Post-zygotic isolation mechanisms comply with Haldane’s rule, with first filial generation (F1) hybrid males being sterile and females, the homogametic sex, being fertile^19–21^. Although a number of studies suggest that hybrids are detected at extremely low frequencies (0.02–0.76%) in areas where *A*. *gambiae* and *A*. *arabiensis* are sympatric, it has recently been pointed out that this could be a large underestimation due to an inefficient method for the identification of hybrids beyond the first generation^22–25^. Given the concrete possibility of gene flow between these species, and more generally between members of the *Anopheles gambiae* complex, carefully designed vector control technologies that function across sibling vector species would be of great interest.

Similarly to *A*. *gambiae*, *A*. *arabiensis* mosquitoes harbour the rDNA repeats, carrying the I-PpoI target site, exclusively in the centromeric region of chromosome X^26–28^. This genetic feature provides the biological requirements to assess whether sex distortion techniques, developed in *A*. *gambiae*, can efficiently be transferred to this important mosquito species. In the present study we describe an experimental set up, based on genetic crosses and transgene selection, that allowed us to introgress the transgenic construct carrying the sex distorter I-PpoI, originally integrated at an autosomal location in the *A*. *gambiae* genome^4^, into the *A*. *arabiensis* genetic background. Fertility of I-PpoI introgressed males and sex bias in their progeny was investigated.

## Results

### Generation of F1 hybrids carrying the sex distorter I-PpoI

**Figure 1:**
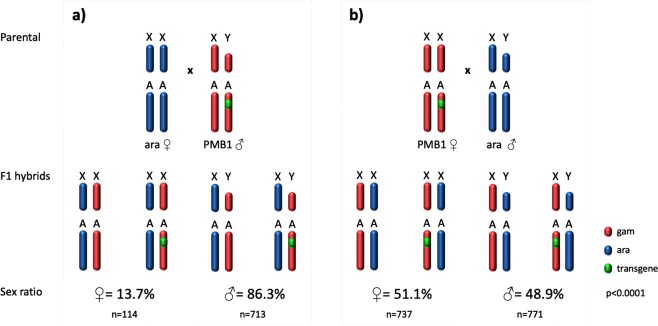
Generation of F1 hybrids in reciprocal crosses between *A*. *arabiensis* and *A*. *gambiae* PMB1 mosquitoes. (**a**) *A*. *arabiensis* females (blue) are crossed to PBM1 males (red) to generate F1 hybrids. The transgene in PMB1 strain, containing the sex distorter I-PpoI and GFP, is highlighted (green dot) in one of the autosomes. (**b**) Reciprocal cross. The parental genetic contribution in the progeny follows the colour code indicated in the legend. Sex ratio values for F1 progeny are indicated as well as the number of females and males counted (n). Significance (p < 0.0001, two-tailed Fisher’s exact test) was tested comparing number of males and females obtained from crosses of *A*. *arabiensis* females and PMB1 males versus number of males and females obtained from crosses of PMB1 females and *A*. *arabiensis* males.

The *A*. *gambiae* strain ^gfp^124L-2, hereafter referred to as Paternal Male Bias 1 (PMB1), carries the sex distorter I-PpoI on chromosome 3R and shows a strong male bias in the progeny (>95%) coupled with a fertility rate that does not differ significantly from wild-type mosquitoes^4^. PMB1 male mosquitoes express both I-PpoI and enhanced green fluorescent protein (eGFP) as in-frame fusion proteins driven by the *β2 tubulin* promoter, specifically active during mosquito male meiosis. An additional marker, 3xP3-RFP gene, allows the identification of transgenics from the early larval stage by virtue of visible RFP expression in the eye and dorsal ganglion. PMB1 mosquitoes were used as the starting strain from which to introgress the autosomal transgene into the *A*. *arabiensis* genetic background and to assess whether this species is susceptible to sex distortion driven by I-PpoI activity. To generate F1 hybrids, we set up *en masse* crosses between *A*. *arabiensis* and *A*. *gambiae* PMB1, heterozygous for the transgene (Fig. 1). Strong male bias (86.3%) was observed in the progeny of transgenic males (Fig. 1a). In the reciprocal cross, where PMB1 females were used, the sex ratio observed in the progeny was in line with Mendelian rules of segregation (~50%) and consistent with the I-PpoI being active only in males (Fig. 1b).

### Reproductive phenotype of F1 hybrid males

**Figure 2:**
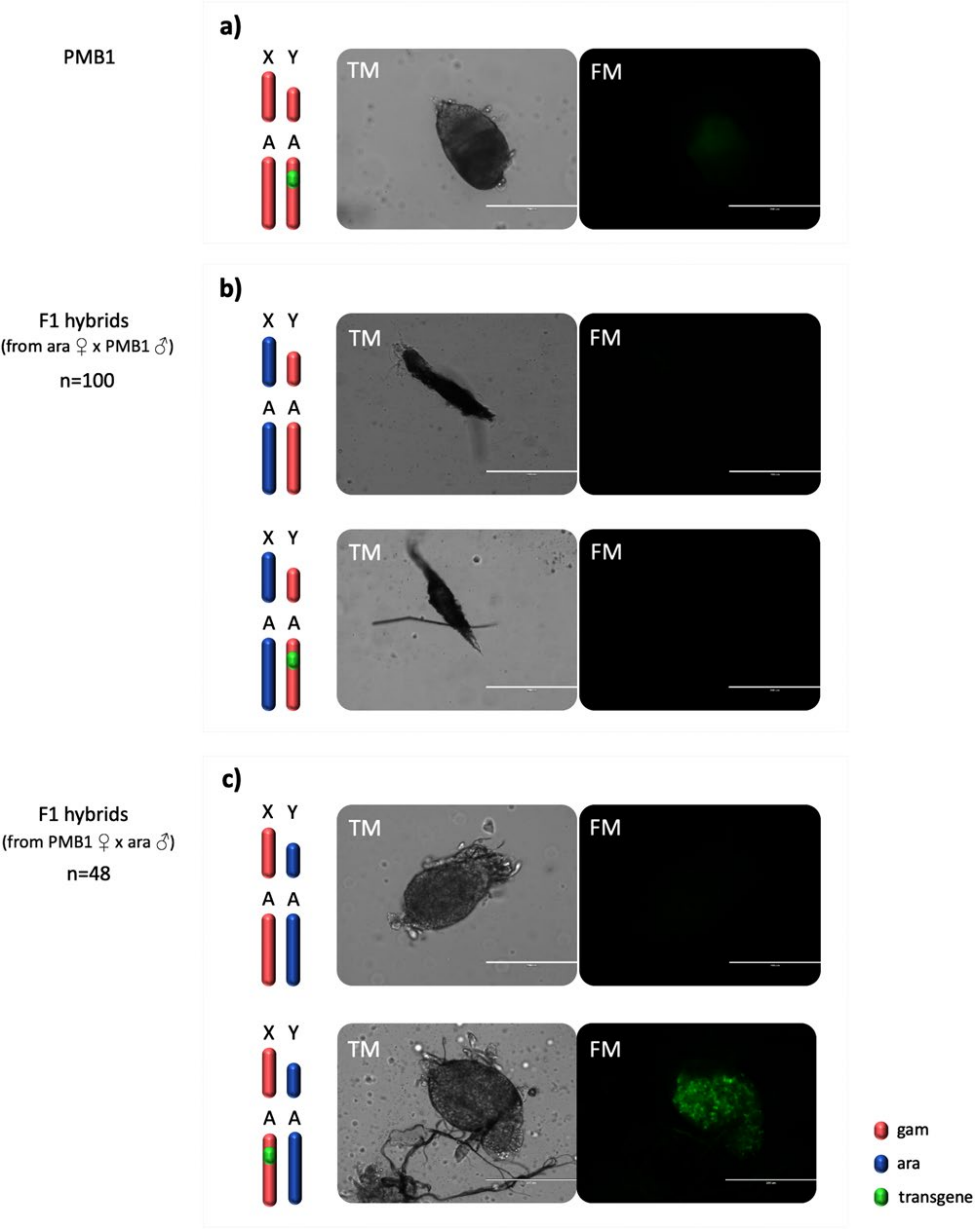
Transmission (TM) and fluorescence (FM) microscopy analysis of testes dissected from PMB1 and F1 hybrid males generated in reciprocal crosses of *A*. *arabiensis* and *A*. *gambiae* PMB1 mosquitoes. (**a**) Testes from PMB1 males looked healthy and expressed the typical β2:GFP decoration pattern along their axis. (**b**) F1 hybrid males generated from PMB1 fathers showed complete organ atrophy and lack of GFP expression. (**c**) F1 hybrid males generated from *A*. *arabiensis* fathers showed normal-shaped testes but lacked mature sperm. A patchy distribution of GFP is observed in testes of transgenic males.

F1 hybrids between *A*. *gambiae* and *A*. *arabiensis* obey Haldane’s rule^19,29^ whereby the heterogametic sex, in this case males, are expected to be sterile. This was confirmed by crossing F1 hybrid males to *A*. *arabiensis* females and recovering 6019 eggs, of which none hatched (data not shown). Analysis of internal reproductive organs revealed that spermatogenesis in all F1 hybrid males was impaired and, consequently, no mature sperm were observed (Fig. 2). Interestingly, ubiquitous atrophy (100/100) was detected when F1 hybrid males were generated from *A*. *arabiensis* mothers and PMB1 fathers (Fig. 2b). Conversely, a less severe morphological phenotype was observed when males were generated from the reciprocal cross, PMB1 mothers and *A*. *arabiensis* fathers. Testes morphology in these males was normal at the macro level (48/48). These testes were clearly recognizable and, in transgenic males, they were populated by cells expressing the GFP marker, thus indicating activity of the meiotic *β2 tubulin* promoter contained within the transgene (Fig. 2c). Nonetheless, the GFP expression in these testes lacked the typical β2:GFP decoration pattern along the testes axis^30^, instead displaying a patchy distribution of GFP and no sign of sperm cell development (Fig. 2).

### Experimental introgression of autosomal I-PpoI from *A*. *gambiae* PMB1 into *A*. *arabiensis*

**Figure 3:**
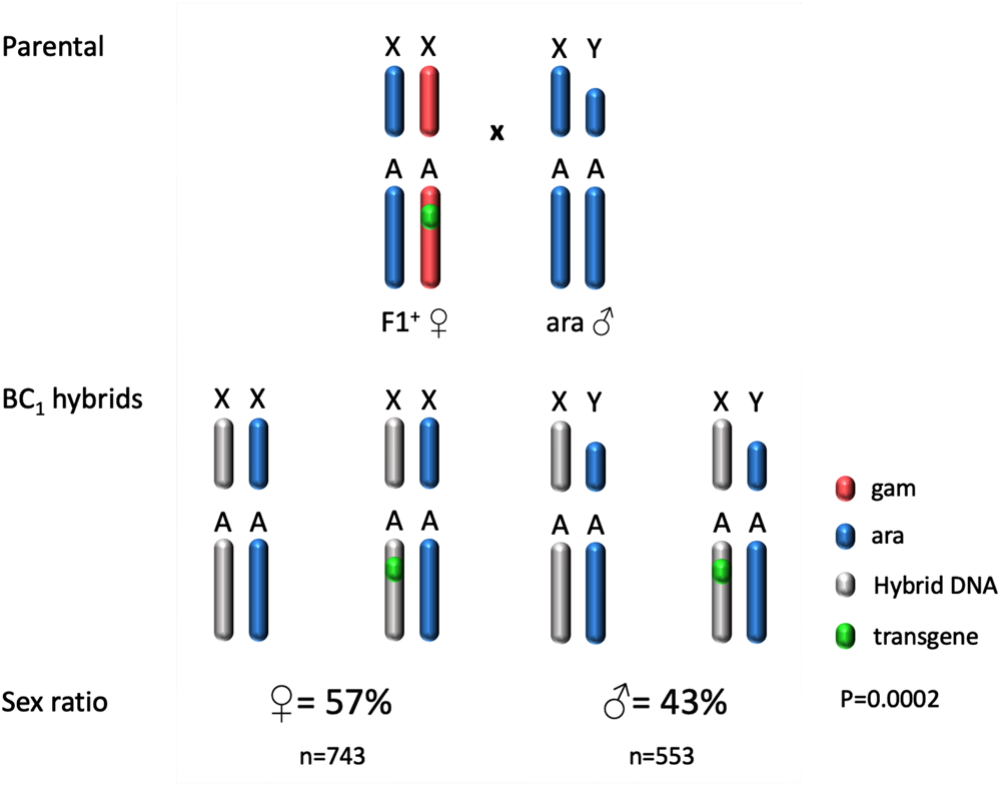
Crossing scheme implemented to generate back cross generation 1 (BC_1_) males. Transgenic F1 hybrid females (blue/red), generated from *A*. *arabiensis* mothers and PMB1 fathers, were crossed to *A*. *arabiensis* males (blue). The autosomal transgene is highlighted (green dot). BC_1_ males inherited *A*. *arabiensis* DNA from their fathers whereas the maternal genetic contribution is the result of meiotic recombination between heterospecific chromosomes (grey). Sex ratio values for BC_1_ progeny are indicated as well as the number of females and males counted (n). Significance (p = 0.0002, two-tailed Fisher’s exact test) was tested comparing number of males and females obtained from crosses of F1^+^ females and *A*. *arabiensis* males versus number of males and females expected according to Mendelian rules of inheritance.

**Figure 4:**
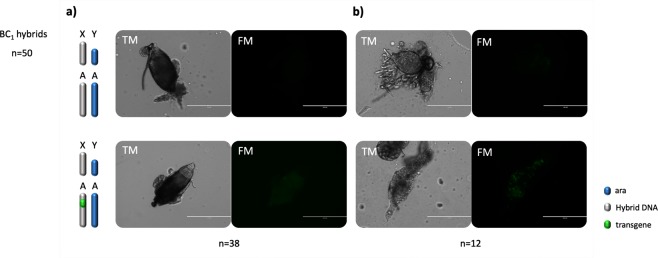
Transmission (TM) and fluorescence (FM) microscopy analysis of testes dissected from BC_1_ males. Organ development and GFP expression were analysed in a total of 50 males. (**a**) The majority (38/50) of testes analysed showed no obvious morphological anomalies and the testes of transgenic males showed a GFP fluorescent signal in line with the transcription pattern of the *β2 tubulin* promoter. (**b**) Testes dissected from 12 males showed undeveloped cells and lack of mature sperm.

In order to progress in the introgression experiment, transgenic F1 hybrid females, generated from crosses between *A*. *arabiensis* females and PMB1 males (Fig. 1a), were selected. These females were mated *en masse* with *A*. *arabiensis* males to generate a backcross (BC_1_) population. The eggs collected from this experiment hatched, generating both male and female mosquitoes. We noticed a small but significant female bias that may be explained by general inviability effects that have occasionally be observed in male hybrids^20^ (Fig. 3). A total of 50 BC_1_ males were randomly selected for testes dissection and analysis. The majority of them (38/50) appeared normal with mature sperm (Fig. 4a), while 12 out of 50 showed undeveloped cells and a lack of mature sperm (Fig. 4b). The observation of normal testes along with β2:GFP expression provided the rationale to carry out an introgression experiment over multiple generations.

**Figure 5:**
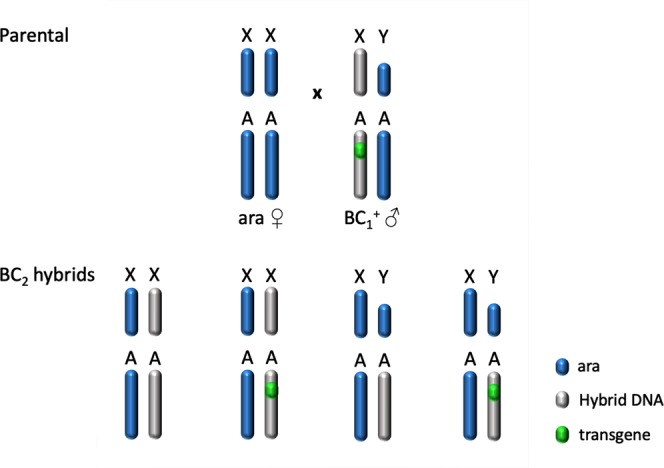
Crossing scheme implemented to generate BC_2_ males. BC_1_ males (blue/grey) were crossed to *A*. *arabiensis* females (blue) to generate BC_2_ males. The autosomal transgene is highlighted (green dot).

Transgenic BC_1_ males were crossed *en masse* to *A*. *arabiensis* females (Fig. 5). After mating and blood-feeding, females were allowed to deposit eggs individually. Only 1 out of 50 females laid eggs that hatched into larvae and a 98% male sex bias was observed in the offspring (data not shown). BC_1_ males carry an X chromosome that might result from heterospecific recombination in the gametes of their F1 hybrid mothers (Fig. 3). Transgenic BC_2_ males, on the other hand, inherit their X chromosome from wild-type *A*. *arabiensis* mothers (Fig. 5a). The presence of this chromosome in combination with the autosomal transgene provides the genetic set up necessary to asses sex distortion in the progeny due to activity of I-PpoI on a ‘pure’ *A*. *arabiensis* X chromosome.

### Effect of I-PpoI on sex ratio distortion in I-PpoI introgressed males

**Figure 6:**
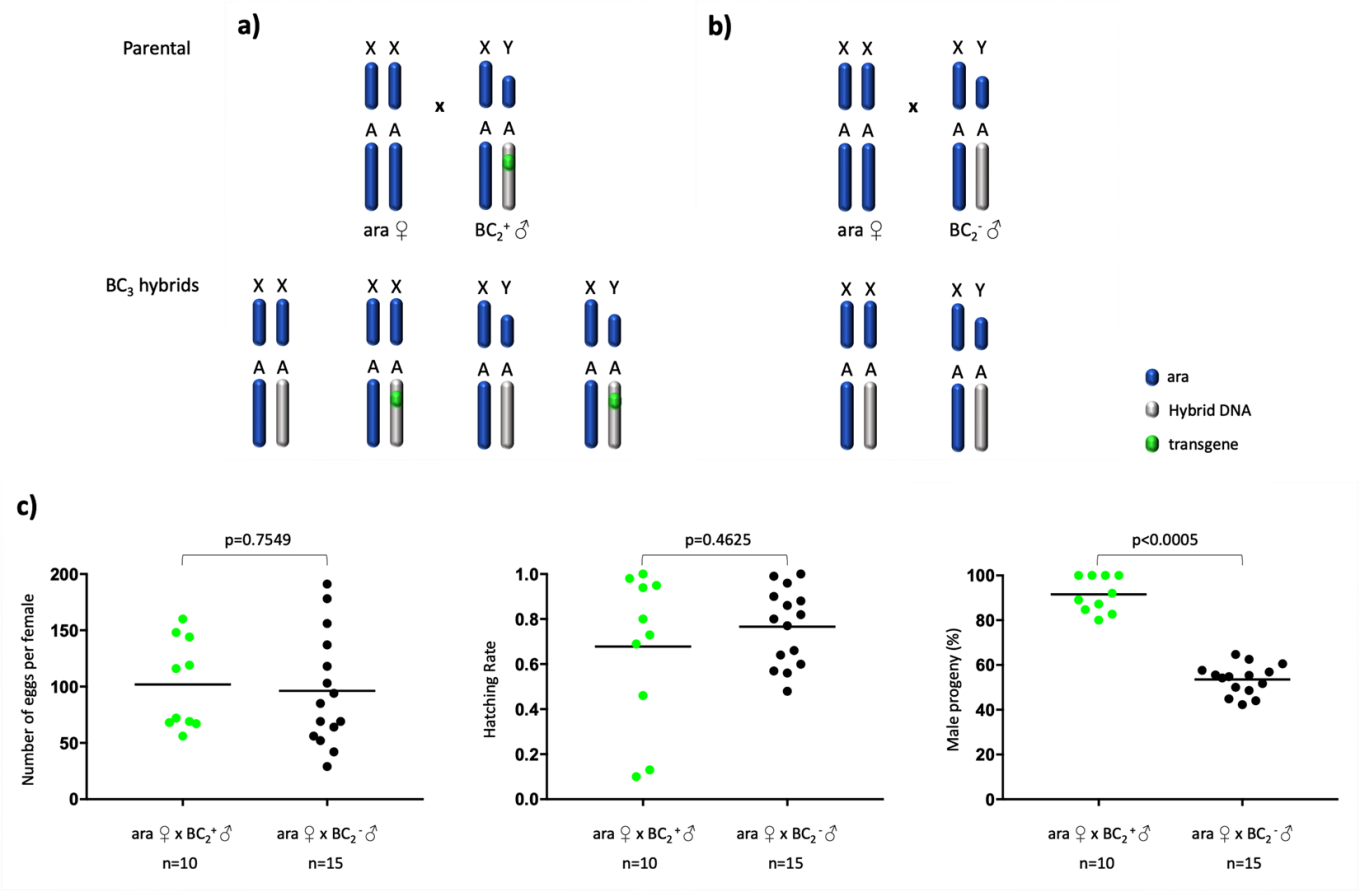
Crossing scheme implemented to generate BC_3_ males. Transgenic (**a**) and non-transgenic (**b**) BC_2_ males (blue/grey) were crossed in single copula mating to *A*. *arabiensis* females (blue) to generate BC_3_ males. The autosomal transgene is highlighted (green dot). (**c**) To assess the fertility of BC_2_ males and to investigate the activity of the sex distorter in the *A*. *arabiensis* genetic background, the oviposition (left panel) and hatching rate (middle panel), as well as the sex ratio in the progeny (right panel), were analysed for each cross. For oviposition, significance (p = 0.7549, two-tailed unpaired t test with Welch’s correction) was tested comparing number of eggs laid by females after mating with ${{\rm{BC}}}_{2}^{+}$ males versus number of eggs laid by females after mating with ${{\rm{BC}}}_{2}^{-}$. For hatching rate, significance (p = 0.4625, two-tailed unpaired t test with Welch’s correction) was tested comparing hatching rate values from progeny of females mated with ${{\rm{BC}}}_{2}^{+}$ males versus hatching rate values from progeny of females mated with ${{\rm{BC}}}_{2}^{-}$. For sex ratio, significance (p < 0.0005, two-tailed unpaired t test with Welch’s correction) was tested comparing number of males from progeny of females mated with ${{\rm{BC}}}_{2}^{+}$ males versus number of males from progeny of females mated with ${{\rm{BC}}}_{2}^{-}$.

**Figure 7:**
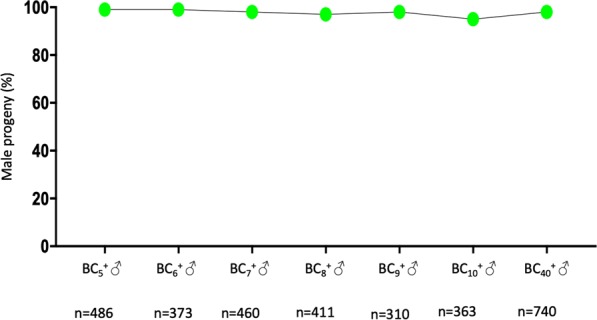
Graph showing the percentage of sex distortion recovered from I-PpoI introgressed males at different backcross generations (from generation BC_5_–BC_10_ and BC_40_).

**Figure 8:**
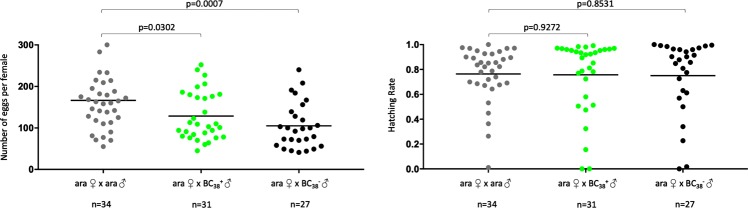
Fertility assay of transgenic and non-transgenic I-PpoI introgressed males (BC_38_). Fertility was assessed in a single copula mating experiment with *A*. *arabiensis* females. Number of eggs laid by individual females and hatching rate are shown on the left and right panel respectively. For oviposition, significance (p = 0.0302 and p = 0.0007, two-tailed unpaired t test with Welch’s correction) was tested comparing number of eggs laid by females after mating with *A*. *arabiensis* males versus number of eggs laid by females after mating with ${{\rm{BC}}}_{38}^{+}$ or ${{\rm{BC}}}_{38}^{-}$ males. For hatching rate, significance (p = 0.9272 and p = 0.8531, two-tailed unpaired t test with Welch’s correction) was tested comparing hatching rate values from progeny of females mated with *A*. *arabiensis* males versus hatching rate values from progeny of females mated with ${{\rm{BC}}}_{38}^{+}$ or ${{\rm{BC}}}_{38}^{-}$ males.

We set up an experiment where transgenic BC_2_ males were used for single copula mating with *A*. *arabiensis* females (Fig. 6a). In a parallel experiment, sibling BC_2_ males that did not inherit the sex distorter were crossed to *A*. *arabiensis* females (Fig. 6b). No significant difference in the number of eggs per female and hatching rate values was observed between the two experimental groups. Interestingly, progeny recovered from crosses of transgenic BC_2_ fathers showed a male bias that ranged from 80 to 100% whereas sex ratio in progeny recovered from crosses of non-transgenic BC_2_ males was in line with Mendelian rules of inheritance (Fig. 6c). Transgenic BC_2_ males from two families were selected, based on high rates of sex distortion and hatching rate, for further introgression, through the male line, of the transgene into the *A*. *arabiensis* genetic background. Values of sex ratio distortion for generation BC_5_–BC_10_ and BC_40_ are shown in Fig. 7. At generation 38, the fertility of I-PpoI introgressed males and their non-transgenic siblings was compared in single copula mating with *A*. *arabiensis* females. In parallel, a control experiment was performed with wild-type *A*. *arabiensis* mosquitoes. The number of eggs per female and hatching rate data for the genetic crosses are shown in Fig. 8. Although a slight difference in the number of eggs per female was observed among the samples analysed, no significant difference in hatching rate values was found between transgenic and non-transgenic introgressed males, or between these males and the wild-type *A*. *arabiensis* males.

## Discussion

*A*. *gambiae* mosquitoes can be genetically engineered to express endonucleases that recognise and cut DNA sequences exclusively present on the X chromosome. When the activity of such enzymes is restricted to spermatozoa development, X-carrying spermatozoa are targeted and a strong bias towards males is induced in the progeny. This strategy has been proven to be effective in *A*. *gambiae* when either the endonuclease I-PpoI or a CRISPR-based nuclease were used^4,9^. Given that reproductive isolation between members of the *Anopheles gambiae* complex is incomplete, it can be assumed that a transgenic construct designed to target *A*. *gambiae* mosquitoes could potentially be transferred to a non-target species within the complex. The strategy described in this work, based on genetic crosses and selection of the marked transgenic effector, was aimed at introgressing the sex distorter I-PpoI from *A*. *gambiae* into the *A*. *arabiensis* genetic background and assessing its capability to produce sex ratio distortion upon recognition and cut of its target sequence on the *A*. *arabiensis* X chromosome. Male bias in the progeny of I-PpoI introgressed males ranged from 95% to 99% throughout the 39 generations that were analysed. In addition, fertility of I-PpoI introgressed males was not significantly different from wild-type *A*. *arabiensis* mosquitoes. Taken together, these results suggest that the I-PpoI-based sex distortion technique can be transferred, in a laboratory setting, between two of the most important members of the *Anopheles gambiae* complex and that this technology works as efficiently in *A*. *arabiensis* as it does in *A*. *gambiae*. These results should inform regulatory and safety assessments regarding the potential implementation of vector control strategies based on sex distortion techniques, and more broadly the development of technologies for wide scale use within the *Anopheles gambiae* complex.

In its current status, the use of sex ratio distortion techniques for field application would require continuous mass releases of mosquitoes to achieve population suppression as the autosomal-linked transgene is inherited by only half of the progeny in every generation. We have previously engineered the *A*. *gambiae* Y chromosome to enable the site-specific insertion of transgenes^31^ and we have successfully introgressed it into the *A*. *arabiensis* genetic background^21^. The insertion of the sex distorter on the male sex chromosome could generate a Y-driving system that, in theory, could spread rapidly and eliminate a target vector population even when a low number of transgenic males is released^2^. The development of such a Y-drive system has been hampered by the inability to express transgenes, under the control of meiotic promoters, from the sexual chromosomes due to Meiotic Sexual Chromosome Inactivation (MSCI)^32^. However, the rapid expansion of molecular tools, coupled with an increased understanding of mosquito biology, raises the prospect of overcoming MSCI and enabling the development of a Y-drive sex distortion system in *A*. *gambiae*. The high level of sex distortion that was achieved in the I-PpoI introgressed strain suggests that such a Y-drive system could potentially be used to target *A*. *arabiensis* mosquitoes.

## Materials and Methods

### Mosquito strains

For these experiments, wild-type *A*. *gambiae* (G3 strain) and wild-type *A*. *arabiensis* (Dongola strain) were used. The G3 strain was isolated from MacCarthy Island in the Republic of the Gambia and obtained from MR4 (MRA-112). The Dongola strain was originally isolated in Sudan and obtained from MR4 (MRA-856). The G3 strain is a hybrid strain containing genetic material derived from both *A*. *gambiae s*.*s*. and *A*. *coluzzii*. The *A*. *gambiae* strain ^gfp^124L-2, referred to in this study as Paternal Male Bias 1 (PMB1), was generated in 2014^4^. The transgenic strain expresses both I-PpoI and enhanced green fluorescent protein (eGFP) as in frame fusion proteins driven by the spermatogenesis-specific *β2 tubulin* promoter. The presence of an additional fluorescent marker, 3xP3-RFP gene, allows the identification of transgenics from the early larval stage by virtue of visible marker expression in the eye and dorsal ganglion.

### Mosquito rearing

For mosquito crosses involving up to 100 mosquitoes, adults were housed in BugDorm-4 insect rearing cages measuring 17.5 × 17.5 × 17.5 cm. Crosses of over 100 adult mosquitoes were kept in BugDorm-1 cages measuring 30 × 30 × 30 cm. Single copula matings were carried out in paper food cups covered with disposable mob caps. Adult mosquitoes were kept in standard conditions at 28 °C and 80% relative humidity with access to 5% (wt/vol) glucose solution for food. After being given 6 days to mate, females were fed either on mice or on bovine blood using a Hemotek membrane feeding system. Two days after the blood meal, an egg bowl containing rearing water (dH20 supplemented with 0.1% pure salt) was placed into the cage. For single deposition experiments, individual females were removed from the cage and placed into cups containing rearing water. For single copula mating, rearing water was added to the individual cups containing the male and the females. On the day of hatching, larvae were transferred to trays containing rearing water and fish food where they were kept until pupation.

### Selection of transgenic mosquitoes

To analyse the expression of eGFP, testes were dissected out of young (<4 days post emergence) adult mosquitoes and analysed on a Nikon inverted microscope (Eclipse TE200) at a wavelength of 488 nm (Filter 535/20 nm emission, 505 nm dichroic). RFP expression was detected in pupae at a wavelength of 563 nm (Filter 630/30 nm emission, 595 nm dichroic).

### Genetic crosses and fertility assay

For the initial parental genetic cross, 50 transgenic PMB1 mosquitoes were crossed to 50 wild-type *A*. *arabiensis* mosquitoes (Fig. 1). F1 hybrids were separated based on sex and the presence of the transgene. To verify F1 hybrid male sterility, 50 transgenic and non-transgenic F1 hybrid males were crossed to 50 wild-type *A*. *arabiensis* females respectively (data not shown). Following oviposition, the eggs were monitored for 4 days for any potential hatching. To progress the introgression, 50 F1 transgenic females were crossed to 50 wild-type *A*. *arabiensis* males (Fig. 3) and the BC_1_ progeny was divided based on sex and fluorescence. To generate BC_2_ progeny, 140 transgenic BC_1_ males were crossed to 100 wild-type *A*. *arabiensis* females (Fig. 5). A total of 87 females were transferred to cups, 50 of which laid eggs. The eggs were monitored for hatching for 4 days, and the progeny resulting from the sole clutch that hatched were separated based on sex and fluorescence. To assess the fertility and I-Ppol activity in BC_2_ males, 23 non-transgenic and 20 transgenic BC_2_ males were placed into individual cups and crossed to 3 wild-type *A*. *arabiensis* females each (Fig. 6). Following mating and a blood meal, rearing water was added to the cups to enable oviposition by the females. The eggs were monitored for hatching for 4 days following oviposition. The number of eggs and larvae was recorded, as well as the sex of the progeny. To continue the introgression, from this point onwards in each generation 50 transgenic I-PpoI introgressed males were selected and crossed to 50 wild-type *A*. *arabiensis* females. The sex bias of the progeny was monitored until generation BC_10_, and then again at generation BC_40_ (Fig. 7). GraphPad Prism software was used to perform statistical analyses in this study. A two-tailed P-value produced by Fisher’s exact test was used for the progeny where sex ratio was analysed. A two-tailed P-value produced by an unpaired t-test with Welch’s correction is reported for the analysis of data about oviposition, hatching rate and % of male progeny.

The protocols and procedures used in this study were approved by the Animal Ethics Committee of Imperial College in compliance with United Kingdom Home Office regulations.
