# Publisher Correction: Introgression of a synthetic sex ratio distortion system from *Anopheles gambiae* into *Anopheles arabiensis*

**DOI:** 10.1038/s41598-019-44277-1

**Published:** 2019-05-22

**Authors:** Federica Bernardini, Antonios Kriezis, Roberto Galizi, Tony Nolan, Andrea Crisanti

**Affiliations:** Department of Life Sciences, Imperial College London, South Kensington Campus, London, SW7 2AZ UK

Correction to: *Scientific Reports* 10.1038/s41598-019-41646-8, published online 26 March 2019

This Article contains errors in the Reference list, where references 4–9 are incorrectly numbered as references 8, 7, 4, 9, 5 and 6 respectively. References 4–9 are correctly numbered below:

4. Galizi, R. *et al*. A synthetic sex ratio distortion system for the control of the human malaria mosquito. *Nat. Commun*. **5**, 3977 (2014).

5. Galizi, R. *et al*. A CRISPR-Cas9 sex-ratio distortion system for genetic control. *Sci. Rep*. **6**, 31139 (2016).

6. Flick, K. E., Jurica, M. S., Monnat Jr, R. J. & Stoddard, B. L. DNA binding and cleavage by the nuclear intron-encoded homing endonuclease I-PpoI. *Nature* **394**, 96 (1998).

7. Jinek, M. *et al*. A programmable dual-RNA-guided DNA endonuclease in adaptive bacterial immunity. *Science* **337**, 816–821 (2012).

8. Windbichler, N. *et al*. Homing endonuclease mediated gene targeting in Anopheles gambiae cells and embryos. *Nucleic Acids Res*. **35**, 5922–5933 (2007).

9. Windbichler, N., Papathanos, P. A. & Crisanti, A. Targeting the X Chromosome during Spermatogenesis Induces Y Chromosome Transmission Ratio Distortion and Early Dominant Embryo Lethality in Anopheles gambiae. *Plos Genet*. **4**, e1000291 (2008).

